# Supplementary material for: Preoperative bedside test indicators as predictors of difficult video laryngoscopy in obese patients: a prospective observational study
Source: PeerJ. 2024 Aug 15;12:e17838. doi: 10.7717/peerj.17838 (PMC11330635; doi:10.7717/peerj.17838)
Supplement: Supplemental Information 3 [file peerj-12-17838-s003.docx]

**Project summary**

The aim of this study was to identify factors associated with difficult video laryngoscopy in obese patients. A total of 579 obese patients undergoing elective laparoscopic weight loss surgery were intubated with a single-lumen endotracheal tube using a video laryngoscopy under general anesthesia, and the patients were divided into two groups based on the Cormack-Lehane classification (difficult video laryngoscopy defined as ≥3): the easy video laryngoscopy group and the difficult video laryngoscopy group. Record the general condition of the patient, bedside testing indicators related to the airway, Cormack-Lehane classification during intubation, and intubation failure rate.

**General information**

**Preoperative bedside test indicators as predictors of difficult video laryngoscopy in obese patients: a prospective observational study**

Liumei Li^1, *^, Guanyu Yang^1, *^, Shiying Li^1^, Xue Liu^1^, Yafei Zhu^1^, Qinjun Chu^1^

^1^ Department of Anesthesiology and Perioperative Medicine, Zhengzhou Central Hospital Affiliated to Zhengzhou University, Zhengzhou, Henan, China

*These authors contributed equally to this work.

Corresponding Author:

Qinjun Chu^1^

Tongbai North Road 16, Zhengzhou, Henan, 450007, China

Email address: 17888551304@163.com

**Rationale & background information**

With the improvement of living standards, the proportion of obese people in China has rapidly increased in the past forty years, and therefore the proportion of obese patients undergoing surgery has also been continuously rising (Chen K et al., 2023). For obese patients who require general anesthesia for surgery, anesthesiologists usually choose tracheal intubation. Airway management in obese patients is relatively challenging because of the accumulation of fat deposits in the airway, thickening of the soft palate and tongue, resulting in a narrow pharynx and severely affecting the exposure of the glottis; in addition, tongue posterior displacement is prone to occur after anesthesia induction, which also affects the exposure of the glottis (Lin CC et al., 2022; Anderson MR et al., 2021; Prathep S et al., 2022). Obese patients typically have increased oxygen consumption and concomitant restrictive lung disease, which reduces the tolerance to failed attempts at tracheal intubation (Jones RL et al., 2006; Bhardwaj S et al., 2019; Mahul M et al., 2016).

One study has shown that the failure rate of tracheal intubation in obese patients can be as high as 15-20% (Higgs A et al., 2018). The laryngoscope is the most commonly used tool for tracheal intubation, and video laryngoscopy has gradually replaced direct laryngoscopy due to its absolute advantages (Hyman JB et al., 2021; Cooper RM et al., 2018; Wu TY et al., 2018). Good vocal cord exposure is the key to success when performing tracheal intubation using a laryngoscope. Previous studies have identified various factors that contribute to difficult laryngoscopy in obese patients, such as body mass index (BMI), Mallampati grade, and neck circumference (Wang T et al., 2018; Mashour GA et al., 2008; Riad W et al., 2016). However, there is limited research on the factors related to difficult video laryngoscopy in obese patients. Therefore, it is important to identify these factors in advance in order to adequately prepare for potential difficult airway. For this purpose, we evaluated the relationship between preoperative bedside testing indicators and the occurrence of difficult video laryngoscopy in obese patients.

**References**

1. Chen K, Shen Z, Gu W, Lyu Z, Qi X, Mu Y, Ning Y. Prevalence of obesity and associated complications in China: A cross-sectional, real-world study in 15.8 million adults. Diabetes Obes Metab. 2023;25(11):3390-3399.

2. Lin CC, Liu KH, Lee LA, Chuang LP, Lin YS, Hsin LJ, Lin WN, Chiang YT, Cheng WN, Li HY. Combined Airway and Bariatric Surgery (CABS) for Obstructive Sleep Apnea Patients with Morbid Obesity: A Comprehensive Alternative Preliminary Study. J Clin Med. 2022;11(23):7078.

3. Anderson MR, Shashaty MGS. Impact of Obesity in Critical Illness. Chest. 2021;160(6):2135-2145.

4. Prathep S, Jitpakdee W, Woraathasin W, Oofuvong M. Predicting difficult laryngoscopy in morbidly obese Thai patients by ultrasound measurement of distance from skin to epiglottis: a prospective observational study. BMC Anesthesiol. 2022;22(1):145.

5. Jones RL, Nzekwu MM. The effects of body mass index on lung volumes. Chest. 2006;130(3):827-833.

6. Bhardwaj S, Garg K, Devgan S. Comparison of opioid-based and opioid-free TIVA for laparoscopic urological procedures in obese patients. J Anaesthesiol Clin Pharmacol. 2019;35(4):481-486.

7. Mahul M, Jung B, Galia F, Molinari N, de Jong A, Coisel Y, Vaschetto R, Matecki S, Chanques G, Brochard L, Jaber S. Spontaneous breathing trial and post-extubation work of breathing in morbidly obese critically ill patients. Crit Care. 2016;20(1):346.

8. Higgs A, McGrath BA, Goddard C, Rangasami J, Suntharalingam G, Gale R, Cook TM; Difficult Airway Society; Intensive Care Society; Faculty of Intensive Care Medicine; Royal College of Anaesthetists. Guidelines for the management of tracheal intubation in critically ill adults. Br J Anaesth. 2018;120(2):323-352.

9. Hyman JB, Apatov D, Katz D, Levine AI, DeMaria S Jr. A Prospective Observational Study of Video Laryngoscopy Use in Difficult Airway Management. Laryngoscope. 2021;131(1):82-86.

10. Cooper RM. Implementing universal videolaryngoscopy: how to do it and what to expect. Br J Anaesth. 2018;120(1):13-15.

11. Wu TY. Education: the last mile to universal videolaryngoscopy. Br J Anaesth. 2018;120(6):1431-1432.

12. Wang T, Sun S, Huang S. The association of body mass index with difficult tracheal intubation management by direct laryngoscopy: a meta-analysis. BMC Anesthesiol. 2018;18(1):79.

13. Mashour GA, Kheterpal S, Vanaharam V, Shanks A, Wang LY, Sandberg WS, Tremper KK. The extended Mallampati score and a diagnosis of diabetes mellitus are predictors of difficult laryngoscopy in the morbidly obese. Anesth Analg. 2008;107(6):1919-1923.

14. Riad W, Vaez MN, Raveendran R, Tam AD, Quereshy FA, Chung F, Wong DT. Neck circumference as a predictor of difficult intubation and difficult mask ventilation in morbidly obese patients: A prospective observational study. Eur J Anaesthesiol. 2016;33(4):244-249.

**Study goals and objectives**

The aim of this study was to identify factors associated with difficult video laryngoscopy in obese patients.

**Study design**

This is a single-center, prospective, observational study approved by the Ethics Committee of Zhengzhou Central Hospital on March 18, 2022 (202236) and registered on March 29, 2022 in the Chinese Clinical Trial Registry (ChiCTR2200058090). All patients had signed a written informed consent before protocol enrollment, in keeping with the Helsinki Declaration. Recruitment of patients scheduled for laparoscopic weight loss surgery under general anesthesia from April 8, 2022 to February 28, 2023. Inclusion criteria: age ≥18 years, BMI ≥28kg/m^2^, American Society of Anesthesiologists (ASA) grade II-III, single-lumen endotracheal intubation using a video laryngoscope. Exclusion criteria: surgery cancelled for various reasons, mental or neurological disorders, patient refusal to participate.

**Methodology**

Collect general information about the patient, including age, weight, height, BMI, sex, comorbidities (diabetes mellitus, rheumatology or rheumatoid arthritis, cervical spondylopathy, neck scar contracture), ASA grades, and dentition (missing, denture). A trained researcher measures the patient's interincisor distance, thyromental distance, neck circumference, assessed the patient's Mallampati grade, and neck movement the day before surgery. This researcher does not participate in the subsequent study process and will keep these results confidential. Table 1 shows the definitions of concepts such as interincisor distance, thyromental distance, neck circumference, Mallampati grade, neck movement, and Cormack-Lehane (C-L) classification, in order to minimize bias as much as possible.

On the day before surgery, all patients underwent a routine preoperative visit by a resident anesthesiologist and signed an informed consent for anesthesia. Perioperative management of the patients was jointly carried out by the resident anesthesiologist and an attending anesthesiologist. The attending anesthesiologist possesses more than 5 years of professional experience. Upon entering the operating room, all patients received standard monitoring (electrocardiography, non-invasive blood pressure, and pulse oximetry). Anesthesia induction was performed using sufentanil 0.5ug/kg (total body weight), propofol 2-2.5mg/kg (ideal body weight), and rocuronium 0.6-1mg/kg (ideal body weight). After 5 minutes, endotracheal intubation was performed by the resident anesthesiologist using a video laryngoscope (TD-C-IV, UE Medical Corporation, Zhejiang, China). The patient's head is positioned in the sniffing position, and a Macintosh size 3 laryngoscope blade (with size 4 available) is used as the standard choice for both male and female patients. The preferred endotracheal tube sizes are 7.0# for females and 7.5# for males (with various sizes available). For patients with a C-L grade of ≥3 during intubation, the following strategies should be attempted in sequence: BURP maneuver (Knill RL et al., 1993), size 4 laryngoscope blade, and a combination of BURP maneuver with a size 4 laryngoscope blade. Alternative options for failed intubation under video laryngoscopy include fiberoptic bronchoscope guidance, laryngeal mask airway, cricothyroid membrane puncture, and tracheostomy. The anesthesiologist performing the intubation records the C-L grade during the video laryngoscopy, whether the BURP maneuver and size 4 laryngoscope blade were used, the success of intubation under video laryngoscopy, and the rescue measures taken after failed intubation under video laryngoscopy on the case report form. Based on the C-L grade (≥3 defined as difficult laryngoscopy), patients are divided into two groups: easy video laryngoscopy group (group E, C-L grade I-II) and difficult video laryngoscopy group (group D, C-L grade III-IV).

**Safety considerations**

Sign informed consent with each patient. Inform of possible risks and measures to be taken. Patients can opt-out at any time. The patient's vital signs were kept stable during the operation.

**Follow-up**

The follow-up observation period is until the patient is discharged from the hospital.

**Data management and statistical analysis**

As an observational study, sample size was not calculated.

Sample size was determined using Gpower 3.1. Based on our preliminary study, the QoR-15 score at 24 hours after preoperative PVB at T8 was 122±12.1 for patients undergoing LSG, while the QoR-15 score at 24 hours after preoperative ESPB at T8 was 129±10.8. Assuming α=0.05, 1-β=0.8, a two-tailed test was conducted, resulting in a calculated sample size of 44 for each group. To account for a potential 20% dropout rate, a total of 110 participants were enrolled in this study.

Use SPSS 23.0 software for data statistical analysis. Normal distribution metric data is represented by mean ± standard deviation, and independent sample t-test is used for between-group comparisons. Non-normal distribution metric data is represented by median and interquartile range, and the Mann-Whitney U test is used for between-group comparisons. Count data is represented as cases (%), and between-group comparisons are performed using chi-square test or Fisher's exact test. Univariate analysis is conducted on potential factors related to the difficult video laryngoscopy, and variables with P<0.05 and variables considered clinically significant are included in the multivariate logistic regression analysis (Forward: Conditional). P<0.05 indicates statistically significant differences.

**Quality assurance**

Studies are carried out in strict accordance with inclusion and exclusion criteria. Try to use objective indicators to evaluate the research results.

**Expected outcomes of the study**

We predicted that interincisor distance and Mallampati grading are correlates of difficult visual laryngoscopy in obese patients.

**Dissemination of results and publication policy**

It is hoped that our results will be published and made available to other centers.

**Duration of the project**

The study is expected to start enrolling patients on April 2022, with an expectation of 11 months. The follow-up observation period is until the patient is discharged from the hospital.

**Problems anticipated**

Withdrawal of the patient and the refusal to participate may occur during the trial. These patients will not be included in the final statistical analysis.

**Project management**

Liumei Li: Conceptualization, Investigation, Methodology, Formal analysis, Writing-original draft. Guanyu Yang: Conceptualization, Formal analysis, Project administration, Supervision, Writing - original draft. Shiying Li: Investigation, Project administration. Xue Liu: Investigation, Project administration. Yafei Zhu: Investigation, Project administration. Qinjun Chu: Conceptualization, Investigation, Formal analysis, Resources, Supervision, Project administration, Writing - original draft.

**Ethics**

The trial protocol will be explained in detail to each patient and informed of what they would participate in, of the possible damage and corresponding measures, eventually enabling each participating patient to sign an informed consent form. This study has been approved by the hospital ethics committee.

**Budget**

The expected cost is mainly the published APC as well as the labor fee of the participants.

**Other support for the project**

This research did not receive any specific grant from funding agencies in the public, commercial, or not-for-profit sectors.
